# Pharmacological Treatment of Alcohol use Disorder in Patients with Psychotic Disorders: A Systematic Review

**DOI:** 10.2174/1570159X21666221229160300

**Published:** 2023-03-09

**Authors:** Niels Jørgen Rosenstand, Anette Søgaard Nielsen, Lotte Skøt, Simon Anhøj, Dorthe Grüner Nielsen, Mikkel Højlund, Angelina Isabella Mellentin

**Affiliations:** 1 Unit for Psychiatric Research, Department of Clinical Research, University of Southern Denmark, Odense, Denmark;; 2 Department of Public Health, Clinical Pharmacology, Pharmacy, and Environmental Medicine, University of Southern Denmark, Odense, Denmark;; 3 Department of Psychiatry, Odense University Hospital, Region of Southern Denmark, Odense, Denmark;; 4 Department of Clinical Research, Brain Research-Inter-Disciplinary Guided Excellence (BRIDGE), University of Southern Denmark, Odense, Denmark;; 5 Department of Psychiatry, Region of Southern Denmark, Svendborg, Denmark;; 6 Drug Treatment Center Odense, Odense C, Denmark;; 7 Department of Psychiatry Aabenraa, Mental Health Services in the Region of Southern Denmark, Aabenraa, Denmark;; 8 Research Unit for Telepsychiatry and E-Mental Health, Center for Telepsychiatry, Region of Southern Denmark, Odense, Denmark

**Keywords:** Pharmacological agents, alcohol use disorder, psychotic disorders, dual diagnosis, side effects, polypharmacy

## Abstract

**Background:**

Patients with psychotic disorders (PD) often have comorbid alcohol use disorder (AUD), which is typically treated pharmacologically. Up till now, no systematic review has examined the effectiveness and safety of AUD treatment in PD patients.

**Objectives:**

This study aimed to systematically review the literature on (1) the effects of pharmacological treatments for AUD on drinking outcomes, (2) the side effects of the drugs, and (3) the effects of polypharmacy in patients with comorbid AUD and PD.

**Methods:**

Bibliographic searches were conducted in MEDLINE, Embase, Cochrane Central Register of Controlled Trials, and PsycINFO. At least two reviewers extracted the data, assessed the risk of bias, and performed the qualitative synthesis of the collected evidence.

**Results:**

Twelve eligible studies were identified, half being randomized controlled trials (RCTs). Three studies examined disulfiram, nine naltrexone, two acamprosate, and one nalmefene by comparing the effects of treatment to placebo, baseline, or pharmacological agents. Disulfiram and naltrexone were shown to reduce alcohol intake. Regarding acamprosate, the findings were mixed. Nalmefene decreased alcohol intake. All pharmacological agents appeared safe to use as AUD monotherapy, but cardiac events were reported when combining naltrexone and disulfiram. Nine studies had a high risk of bias, and three had some other concerns.

**Conclusion:**

The studies provide tentative support for the use of naltrexone and disulfiram in this population, although combinations of pharmacological AUD treatments and other polypharmacy remain unexplored. The studies had high adherence rates that are hardly replicable in real-world settings. Thus, the findings should be confirmed in larger high quality efficacy and effectiveness RCTs with longer follow-ups.

## 1 INTRODUCTION

Alcohol use disorder (AUD) is common among patients with schizophrenia and other psychotic disorders (PD) [1, 2]. Lifetime prevalence rates for AUD among individuals with psychotic disorders have been reported to range between 20% to 34%, and patients with schizophrenia have a more than three-fold increased risk of developing AUD compared to the general population [3, 4]. AUD in patients with schizophrenia has been associated with increasing numbers of hospitalizations, shorter intervals between hospitalizations, and longer lengths of stay [5, 6]. AUD can also exacerbate psychotic symptoms and decrease medication compliance [7, 8].

The standard treatment for AUD in Western countries typically consists of initial sessions of motivational interviewing with subsequent psychotherapy, including cognitive behavioral therapy (CBT), possibly supplemented with pharmacological treatment if the effect of the initial treatment is absent or if the AUD is more severe [9, 10]. The US Food and Drug Administration (FDA) and European Medicines Agency (EMA) have approved disulfiram, acamprosate, and naltrexone for the treatment of AUD [11, 12], and nalmefene and extended-release naltrexone are also approved drugs [12, 13]. The therapeutic effects of pharmacological treatments for AUD are well documented [14], and several more recent meta-analyses on the efficacy and effectiveness of different pharmacological treatments in randomized controlled trials (RCTs) have reported that all the agents showed moderate effects in the treatment of AUD [15-17].

Disulfiram has been shown to be effective in alcohol treatment in open-label studies but not in double-blinded RCTs, as this type of study is not suitable for investigating disulfiram due to its mechanism of action [15]. Acamprosate is often recommended if the goal of treatment is total abstinence, and the drug has been shown to increase the likelihood of remaining completely abstinent [16]. Naltrexone and nalmefene have been found to reduce the desire to consume alcohol. Naltrexone reduces alcohol intake and recurrence, while nalmefene may reduce alcohol intake and heavy drinking [16, 17]. Table **1** summarizes the available pharmacological treatments for AUD [18-24].

Guidelines for patients with comorbid AUD and PD recommend “good practice” based on consensus among colleagues and clinical experience. This applies not only to instructions for psychotherapeutic interventions but also recommendations for pharmacological treatment [25-27]. Using psychotherapy to treat AUD in patients with comorbid schizophrenia or other PD may be unsuccessful due to cognitive impairment and problems of consistent motivation [28-32]. Therefore, pharmacological treatment is an important alternative or supplement to psychotherapeutic treatment for this group of patients, making recommendations essential [32]. This is underlined by the fact that prior research has shown that doctors may be more likely to prescribe the medicine to patients with dual diagnosis compared to those without [33, 34]. This suggests that patients with dual diagnosis are not only particularly vulnerable and refractory towards psychotherapy, but they may also be at risk of polypharmacy, *i.e*., the simultaneous use of multiple drugs [35-37].

To date, very few studies examining pharmacological treatments for AUD have been conducted among patients with comorbid AUD and PD, as they are often excluded from RCTs. Due to the use of other psychotropic drugs and the risk of polypharmacy, side effects and adverse events pose a particular challenge for these patients. Therefore, it is important to gather the scarcely available knowledge about the effectiveness and safety of EMA- and FDA-approved pharmacological treatments for AUD in this vulnerable patient group. However, no systematic review of the available literature has yet been conducted. Hence, the aim of this study was to systematically review the literature on the [1] effects of disulfiram, acamprosate, naltrexone, and nalmefene on drinking outcomes, [2] side effects of the drugs, and [3] effects of polypharmacy in patients with comorbid AUD and PD.

## 2 METHODS

The present systematic review was registered in the Prospective Register of Systematic Reviews (PROSPERO ID: CRD42021273208) and conducted according to recommendations in the Cochrane Handbook for Systematic Reviews of Interventions [38] and the Preferred Reporting Items for Systematic Reviews and Meta-Analyses (PRISMA) statement [39].

### 2.1 Eligibility Criteria

Eligible studies were controlled and uncontrolled intervention studies, including at least one group of patients with AUD and comorbid PD (*i.e*., schizophrenia or schizoaffective disorder) treated with either disulfiram, acamprosate, naltrexone, nalmefene, or combinations thereof, published in peer-reviewed journals. Regarding outcomes, studies that examined changes in drinking outcomes (primary outcomes) were included. Moreover, studies examining changes in psychopathology or craving, side effects, adverse events, or the effects of polypharmacy (secondary outcomes) were included.

### 2.2 Databases and Search Strategy

The following databases were searched: MEDLINE, Embase, Cochrane Central Register of Controlled Trials, and PsycINFO (last search: 3^rd^ July, 2022). Google Scholar was also searched to identify “grey literature”. The full search strategy is provided in Supplementary file S1. Two authors (NJR and AIM) independently screened abstracts from the literature search for full-text reading and later inclusion/extraction, with any disagreements resolved through discussion.

### 2.3 Data Extraction and Data Items

Two authors (NJR and AIM) independently extracted data from the selected studies, with any disagreements resolved through discussion. When possible, the following data were extracted: sample size, study design, study setting (inpatients or outpatients), age, gender and ethnicity of participants, number of cases with PD, number of cases with other psychiatric disorders, abstinence before start of study, pharmacological treatment (including dose), comparison intervention, treatment duration, other interventions, assessment time points, rates of study completion, drinking outcomes, and other outcomes, including psychopathology, craving, side effects (generally expected effects caused to the patient due to intake of the medicine), adverse events (generally unexpected effects caused to the patient due to intake of the medicine), polypharmacy.

### 2.4 Synthesis of Primary and Secondary Outcomes

It was not possible to conduct a quantitative synthesis of the main findings due to the heterogeneity of the study designs and variation in the measurement and/or operationalization of the outcomes. Therefore, a qualitative synthesis and tabulation of the findings from the individual studies were made.

### 2.5 Risk of Bias Assessment

The risk of bias was assessed using either the revised Cochrane Risk-of-Bias Tool for Randomized Trials (RoB 2) [40] or the Risk of Bias in Non-Randomized Studies of Interventions (ROBINS-I) tool [41], depending on the study design. A risk of bias assessment was conducted with respect to the primary and secondary outcomes within the individual studies (across domains). Disagreements between the two reviewers (NJR and AIM) were resolved through discussion.

## 3 RESULTS

### 3.1 Study Selection and Characteristics

**Fig. (1):**
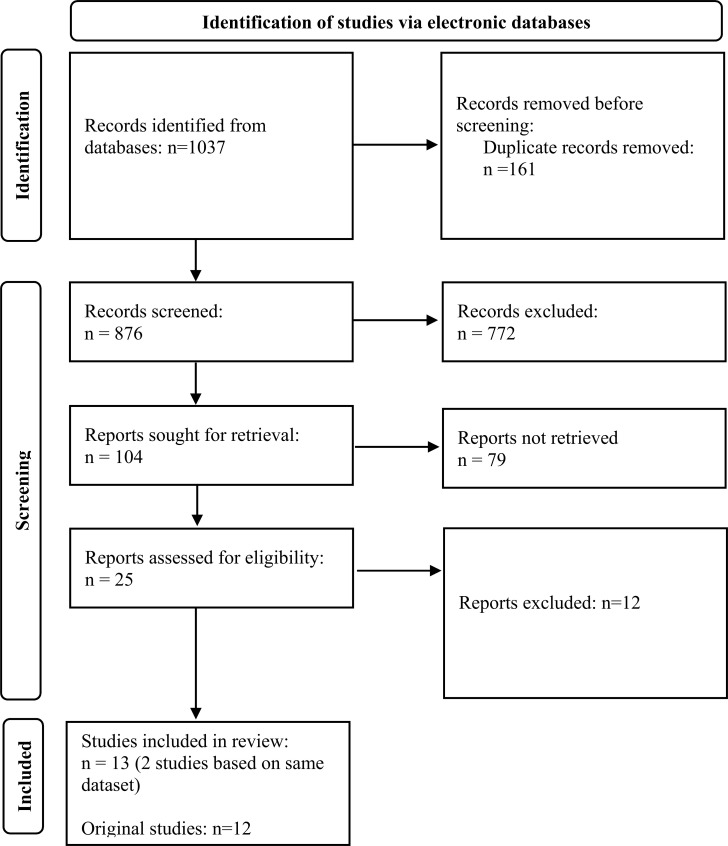
Study selection process.

The database search yielded a total of 1037 records; among them, 876 were unique records (Fig. **1**). The google searches did not produce any additional records. After duplicates were removed, there were 876 records. After screening the titles, 104 records remained. Twenty-five studies were identified after reading the abstracts. Thirteen of these studies (all meeting the criteria for full-text reading) were selected for inclusion. Two of the selected studies were based on the same dataset; therefore, the total number of original studies included in the qualitative synthesis was 12.

Of the 12 original studies, six were either double-blind or open-label RCTs [42-47]. The remaining studies were either open-label, single-arm trials [48-50] or retrospective chart reviews (RCR) [19, 51, 52]. Sample sizes ranged between 12 and 254. Eight studies were conducted with outpatients [19, 42, 44, 45, 47-49, 51], and two studies with inpatients [50, 52]. Two studies did not report whether the patients were inpatients or outpatients [43, 46]. In eight studies, 100% of the patients had a PD (mainly schizophrenia and schizoaffective disorder) [43-46, 49, 50, 52, 53]. In the remaining studies, the percentage of patients with a PD ranged from 7% to 76% (mainly schizophrenia and schizoaffective disorder) [19, 42, 48, 51]. The majority of the studies were conducted with predominately male patients, with percentages ranging from 33% to 100%. Ten studies reported the mean age of the patients, with the average age ranging between 29 and 51 years [19, 42-45, 47-50, 52]. One study reported the age range of the patients, *i.e*., 18 to 65 years [46]. The remaining study did not report the age of the patients [51]. Six studies reported on ethnicity (data not included in Table **2**) [19, 42, 44, 45, 47, 49]. Across these studies, the percentage of Caucasian patients ranged from 35% to 100%. Further characteristics of the included studies can be seen in Table **2**.

### 3.2 Qualitative Synthesis of Primary and Secondary Outcomes

#### 3.2.1 Drinking Outcomes

In a RCR of 33 outpatients, treatment with disulfiram (dose: 150-500 mg/day; treatment duration ranged from less than one month to > 3 years) significantly reduced scores on the Alcohol Use Scale (AUS) at 1-, 2-, and 3-years follow-up compared to baseline (all p-values < 0.001) [19]. Additionally, remission from AUD was observed in 64% of the patients for at least one year during the follow-up period, in 30% for two years, and in 10% for the full three years of follow-up.

In a mixed-blind RCT, 254 outpatients were randomized to receive 12 weeks of either disulfiram alone, naltrexone alone, disulfiram + naltrexone, or placebo (disulfiram dose: 250 mg/day; naltrexone dose: 50 mg/day; disulfiram was dispensed open-label [42]. Overall, patients reduced their alcohol consumption during the trial, and a large proportion remained completely abstinent during the study period (69.7%). At post-treatment, no significant group differences were observed with respect to the percentage of abstinent days, percentage of heavy drinking days, the number of patients with total abstinence, and levels of serum glutamic-oxaloacetic transaminase (SGOT) and serum glutamic pyruvic transaminase (SGPT). Patients treated with either disulfiram or naltrexone had significantly more consecutive days of abstinence (*p <* 0.05) and significantly fewer drinking days per week (*p <* 0.05) at post-treatment compared to the placebo group, but no significant differences were observed between the two active medication groups. Also, disulfiram-treated patients had significantly reduced levels of gamma-glutamyltransferase (GGT) compared to naltrexone-treated patients (*p <* 0.02). The combination of disulfiram and naltrexone had no additional effect on any drinking outcomes compared to either medication alone.

A post-hoc analysis of the mixed-blind RCT described above [42] assessed potential differences between patients with psychotic spectrum disorders (including schizophrenia, schizoaffective disorder, and bipolar disorder; n = 66; hereafter, this unusual classification will be referred to as PD) and those without (n = 185) [53]. Overall, patients with PD had fewer consecutive days of abstinence and total days of abstinence as well as more heavy drinking days than those without PD. Thirty-eight percent of the patients with PD relapsed *versus* 23% of those without (*p* < 0.05). The results also revealed significant interactions between the diagnosis of PD and medication conditions on drinking outcomes. PD patients treated with either disulfiram or naltrexone had significantly more days of abstinence (*p <* 0.01) and fewer heavy drinking days (*p <* 0.02) compared to those who received a placebo. No significant differences in drinking outcomes were observed between PD patients treated with disulfiram and PD patients treated with naltrexone. Furthermore, PD patients treated with both disulfiram and naltrexone did not differ significantly with respect to any drinking outcomes compared to PD patients treated with either active medication alone.

In a RCR of 72 outpatients treated with naltrexone (dose not reported) for eight weeks, reduced drinking was observed at post-treatment compared to baseline [51]. Completers (n = 59; 82%) reduced their drinking by at least 75%, and only 17% of the patients relapsed after eight weeks. In a single-arm, open-label trial [49], 19 outpatients were treated with naltrexone (dose: 350 mg/week) for eight weeks [49]. A significant reduction from baseline to post-treatment was observed with respect to the number of drinks per week (*p <* 0.05), drinks per drinking day (*p <* 0.05), and days of drinking to intoxication (*p <* 0.05). No significant changes were observed with respect to drinking days per week and levels of carbohydrate-deficient transferrin (CDT) and GGT. Another RCR of 12 inpatients treated with naltrexone (dose: 50 mg/day; treatment duration not reported) reported a significant reduction in scores on the AUD identification test (AUDIT) from baseline to [1] time of discharge from hospital and [2] four weeks after hospital discharge (both *p*-values *p <* 0.001) [52].

One single-arm, open-label trial assessed treatment with extended-release naltrexone (dose: 380 mg/every 4 weeks) for 12 weeks in 25 outpatients [48]. Drinking outcomes included drinks per week, drinking days per week, heavy drinking days per week, and drinks per drinking day. The results revealed significant reductions in all drinking outcomes at post-treatment compared to baseline (all p-value < 0.01) values ranging between *p <* 0.01 and *p <* 0.01.

In a double-blind RCT [44], 31 outpatients were randomized to receive naltrexone (dose: 50 mg/day) or placebo for 12 weeks [44]. At post-treatment, the naltrexone group had significantly fewer drinking days (*p <* 0.0001) and heavy drinking days (*p <* 0.01) per week compared to the placebo group. There was no significant drug-by-time interaction effect with respect to drinking days and heavy drinking days. The two groups did not differ significantly from each other with respect to total drinks during the study period. Another double-blind RCT [45] included 90 outpatients randomized to receive naltrexone (dose: 350 mg/week) or placebo for 12 weeks [45]. At post-treatment, the naltrexone group had significantly fewer heavy drinking days compared to the placebo group (*p <* 0.05). However, the two groups did not differ significantly from each other with respect to the number of drinking days and drinks per week.

A 24-week, open-label RCT assessed the effects of naltrexone (dose: 50 mg/day) and acamprosate (dose: 999 mg/day) on drinking outcomes, comparing the agents to each other and counselling alone [46]. The study included 36 patients, but it was not reported whether they were inpatients or outpatients. At post-treatment, both the naltrexone and acamprosate groups had significantly more days of abstinence (*p <* 0.01) and fewer heavy drinking days (*p <* 0.01) compared to the counselling group. A significant reduction in days of abstinence was observed in the acamprosate group compared to the naltrexone group (*p <* 0.01), but the naltrexone group had significantly fewer heavy drinking days than the acamprosate group (*p <* 0.05). Both medication groups also exhibited improved transaminase levels (TL) compared to the counselling group, but the two medication groups did not differ significantly from each other with respect to this outcome.

A 12-week, double-blind RCT randomized 23 outpatients to treatment with acamprosate (dose: about 2 g/day) or placebo. Drinking outcomes included abstinence during the study period, consecutive days of abstinence, drinking days, drinks per drinking day, and heavy drinking days [47]. At post-treatment, the acamprosate group did not differ significantly from the placebo group with respect to any of the outcomes.

Lastly, in a single-arm, open-label trial, 22 outpatients were treated with nalmefene (dose: 18 mg as needed) for 24 weeks [50]. A significant reduction in GGT levels was observed at post-treatment compared to baseline (*p <* 0.001).

#### 3.2.2 Psychopathology, Craving, Side effects, Adverse events, and Polypharmacy

In a RCR examining disulfiram [19], a significant reduction in scores on the Drug Use Scale (DUS) was observed at 1-, 2-, and 3-year follow-up compared to baseline (1 year: *p* < 0.01; 2 years: *p* < 0.05; 3 years: *p* < 0.05) [19, 47]. Also, a significant reduction in the number of days spent in the hospital was observed at 3 years follow-up compared to baseline (*p* < 0.05). An 8-week, single-arm, open-label trial examining naltrexone demonstrated improvements from baseline to post-treatment with respect to scores on the Alcohol Severity Index (ASI; alcohol composite score, *p* < 0.05), the Positive and Negative Syndrome Scale (PANSS; positive scores, *p* < 0.05; negative scores, *p* < 0.01; general psychopathology scores, *p* < 0.01), and alcohol craving (*p* < 0.01) as measured by the Visual Analog Scale (VAS) [49]. Furthermore, in a RCR examining naltrexone (treatment duration not reported), significant improvements were observed from baseline to time of discharge from hospital with respect to overall PANSS scores (*p* < 0.001) as well as scores on the Clinical Global Impressions-Severity Scale (CGI-S; *p* < 0.01), and Global Assessment of Functioning (GAF; *p* < 0.01) [52]. The overall PANSS scores also improved significantly from baseline to four weeks after hospital discharge (*p* < 0.001), and CDSS scores also improved significantly (*p* < 0.01). Furthermore, a single-arm, open-label trial showed that patients administered nalmefene as needed had improved scores on the CGI-S, PANSS, GAF, and Inventory of Drug-Taking Situations – Alcohol-focused version (IDTS-A) at post-treatment compared to baseline [50].

In a 12-week, mixed-blind RCT, disulfiram-treated patients reported significantly lower scores on the Obsessive-Compulsive Drinking Scale (OCDS; total score, *p* < 0.01; compulsive subscale, *p* < 0.01; obsessive subscale, *p* < 0.01) and Brief Symptom Inventory (BSI; obsessive-compulsive subscale, *p* < 0.05; phobic anxiety subscale, *p* < 0.05) at post-treatment compared to naltrexone-treated patients [42]. Also, patients treated with either disulfiram or naltrexone reported significantly lower scores on the OCDS (obsessive subscale, *p* < 0.05) and BSI (paranoid ideation subscale, *p* < 0.05) at post-treatment compared to the placebo group. Moreover, patients treated with a combination of the medications had significantly higher BSI scores (depression subscale, *p* < 0.05) at post-treatment compared to those treated with either medication alone. A 24-week, open-label RCT [43] compared the effects of disulfiram (dose: 250 mg/week) and naltrexone (dose: 50 mg/day) on CGI-S, GAF, IDTS-A, and PANSS scores. The study included 20 patients, but it was not reported whether they were inpatients or outpatients. Both the disulfiram and naltrexone groups [43] exhibited significant improvements from baseline to post-treatment with respect to all the outcomes, but no significant differences were observed between the two groups.

A 12-week, double-blind RCT by Petrakis and colleagues (2004) [44] showed that naltrexone-treated patients had significantly lower scores on the Tiffany Craving Questionnaire (TCQ; total score, *p* < 0.01; desire to drink subscale, *p* < 0.01; intension to drink subscale, *p* < 0.01) at post-treatment compared to the placebo group, while another 12-week, double-blind RCT [45] found no effect of naltrexone on alcohol craving or quality of alcohol high (as measured by the VAS) at post-treatment when compared to placebo. Petrakis and colleagues [44] also reported that PANSS scores at post-treatment did not differ significantly between the naltrexone and placebo groups. In an open-label RCT [46], no significant differences in PANSS, GAF, and IDTS-A scores were observed at post-treatment between patients treated with either naltrexone or acamprosate and the counselling alone group. Furthermore, the two medication groups did not differ significantly from each other with respect to these outcomes. Another 12-week, double-blind RCT [47] showed that treatment with acamprosate did not differ significantly from placebo with respect to scores on the OCDS and PANSS at post-treatment.

Of the three studies examining disulfiram, two reported mild to moderate side effects [19, 43], and one reported mild to severe side effects [42]. Of the studies examining naltrexone, one study did not investigate side effects [45], and one study reported that naltrexone was well tolerated with no severe adverse effects [46]. Other studies reported either mild [44, 51], mild to moderate [43, 49, 52], or mild to severe side effects [42]. Mueser and colleagues reported that seven of the 25 patients who drank while taking disulfiram experienced disulfiram-alcohol reactions, two of which received treatment [19].

In the 2005 mixed blind RCT by Petrakis and colleagues [42], patients treated with either disulfiram or naltrexone as monotherapy were significantly more likely to report mild to moderate side effects when compared to each other or patients who received a placebo (*p*-values ranging between *p <* 0.05 and *p <* 0.01). Moreover, patients treated with a combination of disulfiram and naltrexone were significantly more likely to report mild to severe side effects, including irregular heartbeat, than those treated with either medication alone (p-values ranging between *p <* 0.05 to *p <* 0.01). The post-hoc analysis revealed that patients with PD were significantly more likely to report mild to moderate side effects compared to those without PD (p-values ranging between *p <* 0.05 and *p <* 0.01) [53]. Among patients with PD, there were no differences between the medication groups with respect to side effects.

Petrakis and colleagues (2005) also reported a total of 14 serious adverse events [42]. Among patients treated with a combination of disulfiram and naltrexone, two were hospitalized due to cardiac events, and one experienced a disulfiram-alcohol reaction requiring hospitalization. Among disulfiram-treated patients, four were hospitalized due to psychiatric problems, one had a cardiac event, and one was hospitalized due to acute axonal neuropathy. One naltrexone-treated patient died (death unrelated to the study medication) [53]. In the placebo group, there was one death, one drug overdose, one alcohol overdose, and one hospitalization due to pneumonia. In the post-hoc analysis, it was found that 43% (n = 6) of the 14 adverse events were reported by patients with PD despite the fact that the PD group was a lot smaller than the group without PD [53].

In the double-blind RCT by Petrakis and colleagues [44], two patients in the naltrexone group and one patient in the placebo group were hospitalized due to psychiatric problems, and one patient in the naltrexone group was admitted to the hospital for detoxification. The only study that examined extended-release naltrexone reported mild to moderate side effects as well as one serious adverse event, which worsened psychosis and led to hospitalization [48]. Regarding acamprosate, one study reported that the drug was well tolerated, with no severe adverse events [46]. Another study reported that the side effects of acamprosate did not differ significantly from placebo [47]. Furthermore, one patient in the acamprosate group was hospitalized due to worsening psychiatric symptoms, and another patient visited the emergency department for severe anxiety. However, more adverse events (n = 7) were reported in the placebo group. The only study examining nalmefene reported mild to moderate side effects [50]. In all the studies investigating side effects, it was concluded that the therapeutic benefits of the pharmacological treatment offset any side effects and that the treatment is safe to use during monitoring.

None of the included studies had a design that examined polypharmacy.

### 3.3 Risk of Bias Assessment

The results of the risk of bias assessment are presented in Table **3**. Of the six RCTs, three were rated as having a high overall risk of bias and the other three as having some concerns. The only domains without a high risk of bias rating were attrition and detection bias. In these two domains, one study was rated as having some concerns. Generally, the studies lacked a description of several procedures (*e.g*., generation of random sequence, baseline differences between groups, concealing groups, analyses of outcomes measures). In addition to one study utilizing an open-label design and the fact that none of the studies were pre-registered, it resulted in considerable risk of selection, performance, and reporting bias.

All of the non-randomized studies (n = 6) were rated as having a high overall risk of bias. The only domains without a high risk of bias rating were intervention deviation and intervention classification. In the intervention deviation domain, one study was rated as having some concerns. In the intervention classification domain, all the studies were rated as having some concerns. As was the case with the RCTs, some studies were open-label, and all the studies lacked a description of crucial information, including strategies for reducing confounding, selecting patients and concealing groups, and how missing outcomes were handled statistically. Moreover, none of the studies were pre-registered. Taken together, this resulted in poor scores. In this context, it should be mentioned that three studies were reported in conference abstracts, and therefore it was not possible for the authors to extract exhaustive information.

## 4 DISCUSSION

This systematic review examined the effects of EMA- and FDA-approved pharmacological treatments for AUD on drinking outcomes, the side effects of the medications, and the effects of polypharmacy among patients with comorbid AUD and PD. Twelve studies were eligible for inclusion, six of which were RCTs. The RCTs showed that (1) disulfiram increased consecutive days of abstinence and lowered drinking days per week compared to placebo, (2) naltrexone increased consecutive days of abstinence and reduced drinking days, drinking days per week as well as heavy drinking days compared to placebo, (3) disulfiram reduced GGT levels compared to naltrexone, (4) there was no advantage of combining disulfiram and naltrexone regarding drinking outcomes, and (5) acamprosate had no effect on drinking outcomes compared to placebo. One RCT showed that treatment with acamprosate or naltrexone increased days of abstinence and decreased heavy drinking days and TL compared to counselling alone, with acamprosate having a greater effect than naltrexone on days of abstinence and naltrexone having a greater effect than acamprosate on heavy drinking days. The uncontrolled trials found that treatment with (1) disulfiram reduced AUS scores and increased rates of remission from AUD, (2) naltrexone reduced drinking and lowered drinks per week, drinks per drinking day, and days of drinking to intoxication as well as AUDIT scores, (3) extended-release naltrexone reduced drinking days, drinks per drinking day, heavy drinking days, and drinks per week, and (4) nalmefene administered as needed decreased GGT levels.

Some inconsistent findings regarding drinking outcomes should be noted. Five studies examined the effect of naltrexone on the number of drinking days. While three of these studies found a reduction in drinking days [42, 44, 48], two did not [45, 49]. One possible explanation for the discrepancy is that the latter studies used an unusual dosage compared to ordinary clinical practice (dosing three times weekly instead of daily dosing), which may not have been sufficient to produce an effect. One of the studies used a drug-by-time interaction effect, which presumably makes it more difficult to demonstrate the effect of the drug [44]. Two of the 12 included studies used this analytical approach but failed to demonstrate an effect. One RCT reported an increase in days of abstinence after treatment with acamprosate [46], while another RCT did not [47]. Surprisingly, in the latter study, the dose of acamprosate was higher, and the participants additionally received skills training in relapse prevention, as one would expect a better result. However, all four female patients in the relatively small RCT were randomized to the placebo group, which the researchers point out could have been decisive for the outcome [47].

There was only one study that did not register a decrease in GGT or TL levels, but it did report a decrease in self-reported alcohol use [49]. In addition, the study found no significant association between self-reported alcohol consumption and alcohol biomarkers at baseline, which might be due to reporting bias [49]. In studies on alcohol consumption, biomarkers can be used to validate self-reporting, and underreporting of alcohol consumption is common [54]. In the mixed-blind RCT by Petrakis and colleagues [42], GGT levels decreased significantly in all the groups but no more in the active medication groups (disulfiram, naltrexone, or disulfiram and naltrexone) than in the placebo group. This could be attributed to the fact that there was a large decrease in alcohol consumption in all the groups.

Most of the studies that examined craving and psychopathology (*e.g*., symptoms of psychosis, depression, and anxiety) reported a reduction in symptoms. The RCTs did not report a consistent advantage of pharmacological treatment compared to placebo, which again could be due to the effect of reduced alcohol consumption among all participants. Both studies conducted on inpatients showed a decrease in psychopathological symptoms [50, 52]. Being hospitalized could be an explanation for the improvement in condition. Interestingly, psychopathological symptoms were significantly lowered when monotherapy (disulfiram or naltrexone) rather than combination therapy (disulfiram and naltrexone) was administered [42]. It is possible that the worse side effects observed in the group receiving combination therapy contributed to less improvement in psychopathological symptoms.

Disulfiram, in combination with alcohol, has the greatest potential for inducing side effects. Therefore, disulfiram has traditionally been used when the goal of treatment is complete abstinence. It may be complicated to identify non-drinking-related side effects and adverse events beyond the known alcohol-disulfiram reaction among outpatients since they are not always supervised. Although patients drank while medicated with disulfiram, particularly in one study (76%) [19], only one hospital admission due to alcohol/ disulfiram reaction was reported [42]. There were no cases of disulfiram-induced psychosis. The inhibition of dopamine beta-hydroxylase may induce this condition; however, many of the case reports are from before 1970, when the disulfiram dose used was generally higher. Recent studies have suggested that it is not a clinically significant problem [55]. The studies identified in the present review used low doses of 250 mg of disulfiram and found that disulfiram-induced psychosis did not occur in patients with schizophrenia or schizoaffective disorder. One study even reported a reduced level of paranoid thoughts, and the effect was similar in the naltrexone and disulfiram groups [42]. The reduction may, however, be due to reduced alcohol intake or the use of antipsychotics. However, regarding the latter, neither type(s) nor dose of antipsychotics were reported in the included studies. The risk of consuming alcohol while taking disulfiram should be carefully considered, and caution should be exercised when administering the drug. To address the specific issues associated with disulfiram, studies have indicated that the drug is more suitable for supervised use [56-58].

Studies investigating side effects and adverse events indicated that naltrexone is safe to use. Unlike disulfiram, naltrexone does not cause severe reactions when alcohol is consumed. Complete abstinence may be but is not necessarily a realistic goal, as inconsistent motivation and cognitive challenges complicate abstinence. An alcohol treatment offer that does not require complete abstinence is important for the group with PD. Since using naltrexone does not require complete abstinence, this may increase motivation to start treatment. Acamprosate has the potential to promote complete abstinence without causing the potential problems associated with disulfiram. Moreover, patients receiving acamprosate do not need to be followed as closely. However, compliance issues must be considered as acamprosate has to be taken three times daily.

Nalmefene has the same potential as naltrexone to be used when the treatment goal is alcohol reduction instead of abstinence. Severe side effects were not reported when nalmefene was administered as needed [50], but a recent case-report study [59] reported that one patient with schizoaffective disorder experienced an exacerbation of psychotic symptoms. The symptoms subsided two days after discontinuation of nalmefene. The above findings regarding side effects and adverse events indicate that it is not necessarily straightforward to decide which type of pharmacological treatment will be preferable for the individual patient. It will be a clinical judgment with many factors to consider, such as treatment goal, patient profile, motivation, possibilities for monitoring the patient, other comorbid substance use disorders, and risk assessment.

One of the aims of this study was to assess the problems associated with polypharmacy among AUD patients with comorbid PD who, as a starting point, are often prescribed many psychotropic drugs, including antipsychotics. Antipsychotics have been shown to be useful in alcohol treatment for patients with PD [60], with naltrexone perhaps even exerting a synergistic effect [61]. Since patients in the majority of included studies were prescribed antipsychotics, it was not possible to isolate the effects of specific compounds. However, one study noted no difference in side effects between the group taking one psychotropic drug and the group taking more than one [42]. In general, the low number of reported side effects and adverse events indicate that it is safe to combine drugs targeting AUD and PD, but further research is needed to validate this conclusion.

All the included studies reported high completion rates, and the most crucial factor for the outcome of pharmacological interventions for AUD has, not surprisingly, been compliance [18, 62]. The included studies excluded the most unstable patients and might therefore represent the most stable and compliant segment of the population, thus compromising external validity. High levels of abstinence may explain why studies failed to demonstrate a drug-by-time interaction effect [44, 45]. Petrakis and colleagues refer to this as a “ceiling effect” [44]. When abstinence is generally high in a sample, it becomes difficult to demonstrate an effect of the drug on reduction parameters and especially on complete abstinence. High levels of reported abstinence prior to starting treatment may have impacted drinking outcomes in several studies, including the RCT that examined acamprosate and found no effect of the drug [47].

Regarding characteristics of the participants, males comprised approximately 81% of participants in the included studies, but there were no indications that patients with comorbid AUD and PD responded differently to pharmacological treatments for AUD depending on gender [63, 64]. Three studies were conducted on war veterans [42, 44, 47]. It is not certain whether this group responds in the same way to pharmacological treatment as other groups since research has shown that military personnel have different personality profiles [65-67] and a higher risk of developing post-traumatic stress disorder and other psychiatric disorders [68] compared to the general population. Most patients in the included studies were of Caucasian descent, while African Americans comprised the second largest group, with one study reporting a rate of 61% [47]. Studies examining naltrexone have shown a reduced effect of the drug among African Americans [69, 70]. Thus, the distribution of ethnic groups in the included studies might have impacted drinking outcomes. In general, genetic factors involved in alcohol metabolism have been associated with AUD [71], and AUD treatment outcomes with naltrexone have been linked to specific genotype variants in alcohol-metabolizing enzyme genes [72].

## 5 LIMITATIONS

The studies are difficult to compare due to several factors. The study designs are different, either double-blind/ open-label RCTs, single-arm trials, or RCRs, which have been placed on different levels in the evidence hierarchy. Moreover, several studies were presented in conference abstracts, making some information unclear or inaccessible and generally complicating the process of critical methodological review. Out of the 12 studies, a total of four did not include a sample comprising 100% PD patients. It should also be highlighted that one study did not report the dose of medication and another the treatment duration. Furthermore, the majority of studies assessed alcohol consumption through self-report, and it is well-documented that patients typically underreport their consumption [54]. Moreover, the way in which outcomes were measured varied greatly across the studies; for example, alcohol craving was measured by VAS, TCQ, OCDS, or undisclosed method. Several studies required patients to abstain for a certain period of time before the start of the study, and in other studies, patients were allowed to drink up to the start. Whether or not patients were required to be abstinent before the study may have impacted their ability to abstain. Some of the studies provided patients with additional advice on alcohol detoxification; however, the effect of doing this is unclear [44, 45, 47, 48]. Moreover, the majority of the RCTs included in this systematic review had a follow-up time of max. 12 weeks. It may take longer for the pharmacological agents to work effectively among those with PD [73]. However, studies have shown that in populations without PD, there was no difference in drinking behaviour after 12 months between the naltrexone and placebo groups [74]. Hence, the effects of the pharmacological agents should be tested over longer periods of time in future studies.

## CONCLUSION

This is the first systematic review of the effectiveness and safety of EMA- and FDA-approved pharmacological treatments for AUD among patients with PD. The findings indicated that naltrexone and disulfiram have a beneficial effect on drinking outcomes in patients with comorbid AUD and PD. Acamprosate showed divergent results, and nalmefene decreased alcohol intake, albeit based on limited evidence. All pharmacological agents appeared safe to use as AUD monotherapy, but cardiac events were reported when combining naltrexone and disulfiram. There are several unexplored areas, such as the effects of the drugs over time in more unstable PD patients, as well as the effects of polypharmacy. There are currently not enough studies to provide strong evidence-based recommendations for pharmacological treatments for AUD in patients with comorbid AUD and PD. RCTs examining the effects of pharmacological treatments over longer periods of time are thus greatly warranted for assessing higher-quality efficacy and effectiveness.

## Figures and Tables

**Table 1:** Pharmacological treatments for alcohol use disorder.

**Type of Medication**	**Description of Medication (with Additional Information Applicable to Psychotic Disorder)**
Disulfiram	Disulfiram inhibits acetaldehyde dehydrogenase and thereby accumulates acetaldehyde in the body when alcohol is consumed, and possible side effects include nausea, vomiting, headaches, and tachycardia [18]. The drug has a deterrent effect when alcohol is consumed. The use of disulfiram requires complete abstinence, which can be a challenge for some patients, especially if they also have a psychotic disorder [19]. Since the drug inhibits dopamine beta-hydroxylase, it has the potential to trigger or exacerbate psychosis [20].
Acamprosate	The mechanism of action of acamprosate has not been exhaustively investigated, but there is some evidence that acamprosate modulates the N-Methyl-D-aspartate receptor and upgrades GABA by various mechanisms, such as increased binding in the thalamus and hippocampus [21]. This compensates for the neurobiological disorder during abstinence [21], and the likelihood of continued abstinence is increased.
Naltrexone	Naltrexone, the mechanism of action of which has also not yet been fully elucidated, is a µ- and to a lesser extent ĸ-opioid receptor antagonist that is thought to reduce craving and the feeling of satisfaction from drinking through inhibition of alcohol-released endorphins [22]. The use of naltrexone requires that care be taken if there is concomitant opioid intake or abuse. Polysubstance abuse is common among patients with psychotic disorders; therefore, caution is warranted in the use of naltrexone in this group.
Nalmefene	The opioid receptor ligand, nalmefene, has a full antagonistic effect on the µ-opioid receptor and partly on the δ-opioid receptor, as well as a partly agonistic effect on the ĸ-opioid receptor [23]. Nalmefene has an effect similar to naltrexone, where the effect of endorphins is inhibited, but the drug has a broader profile of action on opioid receptors. This particular effect has given rise to the use of nalmefene as needed, which may provide opportunities in the group with psychotic disorders that may have compliance challenges [24].
Extended-release naltrexone	Extended-release naltrexone is an injectable prolonged-release formulation of naltrexone, giving it potential for use when there are challenges with compliance.

**Table 2:** Study characteristics.

**Study (Sample Size)**	**Study Design and Setting**	**Percentage of Male Participants**	**Mean Age (SD) of the Participants**	**Number (%) of Cases with a Psychotic Disorder**	**Number (%) of Cases with Other Psychiatric Disorders**	**Abstinence before the Start of the Study**	**Intervention Group(s)**	**Other Interventions**	**Treatment Duration**	**Assessment Time Points**	**Drinking Measures**		**Drinking Outcomes**	**Other Outcomes**	**Completion Rate**	**Results**	
											**Self-Report**	**Bio-markers**					
**Disulfiram**																
Mueser *et al*., 2003 [19](N = 33)	RCROutpatients	64%	37 years (± 7.1)	N = 25 (76%)(S: n = 15; SAD: n = 8;UP: n = 1; SPD: n = 1)	N = 8 (24%)(BD: n = 5;MDD: n = 3)	Not reported	Disulfiram 125-500 mg/day	AP + OPTNFS	< 1 month (n = 4);1-3 months (n = 7); 3-6 months (n = 3); 6-12 months (n = 3); 1-2 years (n = 8); 2-3 years (n = 3): > 3 years (n = 8)	1-, 2-, and 3-years following initiation of disulfiram treatment	AUS	None	AUS, remission from AUD	DUS, number of days spent in hospital (per year), number of weeks worked (per year), side effects.	100%(52 weeks)	Reduced AUS scores were observed at 1-, 2-, and 3-years follow-up compared to baseline.Remission from AUD was seen in 21 patients (64%) for at least one year during the follow-up period, in 10 patients (30%) for two years, and in 3 patients (10%) for the full three years of follow-up.Reduced DUS scores were observed at 1-, 2-, and 3-years follow-up compared to baseline.A reduction in the number of days spent in the hospital was observed at the 3-year follow-up compared to baseline.Mild to moderate side effects were reported by 7 patients. Alcohol-disulfiram reactions were experienced by 7 patients.
**Disulfiram *vs*. Naltrexone**																
Petrakis *et al*., 2005, 2006 [42, 53](N = 254)	DB/OL RCT (DB naltrexone/OL disulfiram)Outpatients	97%	47 years (± 8.2)	N = 18 (7%)(S/SAD: n = 18)	MDD: n = 178 (70%)PTSD: n = 109 (43%)GAD: n = 57 (20%)CUD: n = 50 (22%)BD: n = 49 (19%)	min. 3 days; max. 29 days	Naltrexone50 mg/day (n = 59)* vs.*Disulfiram 250 mg/day (n = 66)* vs.*Naltrexone 50 mg/day+ disulfiram 250 mg/day (n = 65)* vs.*Placebo (n = 64)	AP + OPTNFS	12 weeks	Week 12	TLFB	GGTSGOTSGPT	CDA, DA, DDW, HDD, TA, GGT, SGOT, SGPT	OCDS, BSI, side effects (HCSL)	89%	Overall, patients treated with either disulfiram or naltrexone had more CDA and fewer DDW post-treatment compared to the placebo group. Disulfiram-treated patients had reduced levels of GGT at post-treatment compared to naltrexone-treated patients. Patients with PD (S, SAD, BD) had fewer CDA and more HDD than those without PD. Also, patients treated with either disulfiram or naltrexone had more DA and fewer HDD compared with a placebo.Overall, disulfiram-treated patients had lower OCDS and BSI scores at post-treatment compared to naltrexone-treated participants.Patients treated with either disulfiram or naltrexone had lower OCDS and BSI scores post-treatment compared to the placebo group.Overall, there was no advantage of combining disulfiram and naltrexone with respect to any of the outcomes. This was also the case among patients with PD.Mild to severe side effects. Symptoms potentially related to side effects were reported by 96.9% of the participants. Patients with PD were more likely to report mild to moderate side effects compared to those without PD.Fourteen adverse events were recorded, 3 in the combined disulfiram and naltrexone group, 6 in the disulfiram group, 1 in the naltrexone group, and 4 in the placebo group. 43% (n = 6) of the adverse events were reported by patients with PD.
Vasile *et al*., 2013 [43]*(N = 20)	OL RCTSetting not reported	45%	40 years (SD not reported)/	N = 20 (100%)(S: n = 20)	None	Not reported	Naltrexone 50 mg/day (n = 8)* vs.*Disulfiram 250 mg/day (n = 12)	AP	24 weeks	Week 24	None	None	None	CGI-S, GAF, IDTS-A, PANSS, side effects	Max. 75%	Both the disulfiram and naltrexone groups exhibited improvements from baseline to post-treatment on all outcomes, with no differences observed between the two groups.Mild to moderate side effects were observed in both the disulfiram (6 patients) and naltrexone (4 patients) groups.
**Naltrexone**																
Maxwell and Shinder-mann 2000 [51](N = 72)	RCROutpatients	71%	Not reported	N = 24 (33%)S: n = 17(23.3%)SAD: n = 7(5.5%)	MDD: n = 37(50.7%)BD: n = 11(15.1%)GID: n = 4(4.1%)	Not required	Naltrexone unknown dose	OPTNFS^2^	8 weeks	week 8	Not reported	None	Reduced drinking	Side effects	82%	Reduced drinking was observed at post-treatment compared to baseline. Completers reduced their drinking by at least 75%, and only 17% of the patients relapsed after 8 weeks.Mild side effects.
Batki *et al*., 2007 [49](N = 19)	Single-arm, OL trialOutpatients	79%	43 years (±12)	N = 19 (100%)(S: n = 11; SAD: n = 7; UP: n = 1)	None	Not required	Naltrexone 350 mg/ week	AP + OPTNFS	8 weeks	week 4week 8	TLFB	CDTGGT	DDW, DWDDD, DDI% CDT% GGT	ASIVAS (craving and VAS QoH), PANSS, side effects	74%	A reduction in DW, DDD, and DDI, as well as in scores on the ASI, VAS, and PANSS, was observed at post-treatment compared to baseline.No associations were found between self-reported drinking outcomes and alcohol biomarkers.Mild to moderate side effects.
Batki *et al*., 2010 [48]*(N = 25)	Single-arm, OL trialOutpatients	52%	44 years (SD not reported)	N = 17 (68%)(S :n = 6; SAD: n = 11)	N = 8(BD: n = 8)	Not reported	Extended-release naltrexone 380 mg/ q4wk	AP + PI (motivational counselling- weekly sessions)	12 weeks	week 12	TLFB (outcome not reported)	None	DW, DD, HDD, DDD	Side effects	84%	Significant improvements were observed from baseline to post-treatment with respect to all outcomes.Mild to moderate side effects. One serious adverse event was recorded.
Vasiliu *et al*., 2020 [52]*(N = 12)	RCRInpatients	33%	29 years (SD not reported)	N = 12 (100%)(SAD-bipolar type: n = 2; SAD-depressive type: n = 10)	None	min. 10 days	Naltrexone 50mg/day	AP	Not reported	At discharge from hospital 4 weeks after hospital discharge	AUDIT	None	AUDIT score	CGI-S, CDSS, GAF, PANSS, side effects	Not reported	AUDIT and PANSS scores improved from baseline to (1) the time of discharge from the hospital and (2) four weeks after hospital discharge. CGI-S and GAF scores improved from baseline to time of discharge from hospital. CDSS scores improved significantly from baseline to four weeks after hospital discharge.Mild to moderate side effects.
Petrakis *et al*., 2004 [44](N = 31)	DB RCTOutpatients	100%	46 years (± 5.7)	N = 31 (100%)(S: n = 18; SAD: n = 13)	None	Not required	Naltrexone 50 mg/day (n = 16)* vs.*Placebo (n = 15)	AP + PI (cognitive behavioral relapse prevention strategies – weekly sessions)	12 weeks	week 12	TLFB (used to validate UTS but outcome is not reported)	UTS	DD, HDD, TDSP	TCQ, PANSS, side effects (HSCL)	81%	A reduction in DD and HDD at post-treatment was observed in the naltrexone group compared to the placebo group. There was no drug-by-time interaction effect with respect to either DD or HDD. A reduction in TCQ scores at post-treatment was observed in the naltrexone group compared to the placebo group.Mild side effects. A total of 4 adverse events were recorded, 3 in the naltrexone group and 1 in the placebo group.
Batki *et al*., 2009 [45]* (N = 90)	DB RCTOutpatients	71%	42.5 years (SD not reported)	N = 90 (100%)(S: n = 45; SAD: n = 45)	None	Not reported	Naltrexone 350 mg/week (n = 45)* vs.*Placebo (n = 45)	PI (motivational counselling- weekly sessions)	12 weeks	week 12	TLBF (outcome not reported)	None	DW, DD, HDD	VAS (craving and QoH)	Not reported	At post-intervention, a reduction in HDD was observed in the naltrexone group compared to the placebo group.Side effects were not examined.
**Naltrexone * vs*. Acamprosate**																
Bratu and Sopterean 2014 [46]* (N = 36)	OL RCTSetting not reported	67%	Mean age not reportedAge range: 18-65 years.	N = 36 (100%)(S: n = 36)	None	min. 7 days	Naltrexone 50 mg/day (n = 12)* vs.*Acamprosate 999 mg/day (n = 12)* vs.*Counselling (n = 12)	AP	24 weeks	Week 24	Not reported	TL	DA, HDD, change in TL	GAF, IDTS-A, PANSS, side effects	Not reported	At post-treatment, an increase in DA and a reduction in HDD were observed in both medication groups compared to the counselling group. An increase in DA was observed in the acamprosate group compared to the naltrexone group, but the naltrexone group had fewer HDD than the acamprosate group. Both medication groups also exhibited improved TL scores compared to the counselling group.Both naltrexone and acamprosate treatment were well tolerated, with no severe adverse effects.
**Acamposate**																
Ralevski *et al*., 2011 [47] (N = 23)	DB RCTOutpatients	83%	51 years (±7.3)	N = 23 (100%)(S: n = 9, SAD: n = 10; UP: n = 4)	None	min. 5 days; max. 21 days	Acamprosate 1998 mg/day (n = 12)Placebo (n = 11)	AP + PI (skills training in relapse prevention)	12 weeks	Week 12	TLFB	None	ASP, CDA, DD, DDD, HDD	OCDS, PANSS,side effects	65%	At post-intervention, the acamprosate group did not differ significantly from the placebo group with respect to any of the outcomes (including side effects).No differences in side effects were observed between the two groups.A total of 16 adverse events were recorded, 7 in the placebo group (2 events reported by the same participant), 2 in the acamprosate group, and 7 events were deemed unrelated to study participation.
**Nalmefene**																
Vasile *et al*., 2014 [50]*(N = 22)	Single-arm, OL trialInpatients	45%	44 years (SD not reported)	N = 22 (100%)(S: n = 22)	None	Not required	Nalmefene 18 mg pro necessitate	AP	24 weeks	Week 24	None	GGT	Change in GGT	CGI-S, GAF, IDTS-A, PANSS, side effects	82%	A reduction in GGT levels, along with improved scores on the CGI-S, GAF, IDTS-A, and PANSS, were observed at post-treatment compared to baseline.Mild to moderate side effects (12 patients).

**Abbreviations:** AP: antipsychotics; ASI: addiction severity index; ASP: abstinence in study period; AUD: alcohol use disorder; AUDIT: alcohol use disorder identification test; AUS: alcohol use scale; BP: bipolar disorder; BSI: brief symptom inventory; CDA: consecutive days abstinent; CDT: carbohydrate-deficient transferrin; CDSS: Calgary Scale for Depression in Schizophrenia; CGI-S: clinical global impressions - severity; CUD: cocaine use disorder; DA: days abstinent; DB: double-blind; DD: drinking days; DH: days hospitalized; DDD: drinks per drinking day; DDI: days of drinking to intoxication; DDW: drinking days per week; DUS: drug use scale; DW: drinks per week; GAD: generalized anxiety disorder; GAF: global assessment of functioning; GGT: gamma-glutamyltransferase; GID: gender identity disorder; HDD: heavy drinking days; HSCL: Hopkins Symptom Checklist; IDTS-A: inventory of drug taking situations – alcohol focused version; MDD: major depressive disorder; OCDS: obsessive compulsive drinking scale; OL: open-label (unblinded); OPTNFS: outpatient psychiatric treatment not further specified; PANSS: positive and negative syndrome scale; PI: psychological intervention; PTSD: post traumatic stress disorder; QoH: quality of alcohol high; RCR: retrospective chart review; RCT: randomized controlled trial; RD: reduced drinking; S: schizophrenia; SAD: schizoaffective disorder; SGOT, serum glutamic-oxaloacetic transaminase; SGPT: serum glutamic pyruvic transaminase; SPD: schizoid personality disorder; TA: total abstinence ; TCQ: Tiffany Craving questionnaire; TDSP: total drinks in study period; TL: transaminase level; TLFB: Timeline Followback; UP: unspecified psychosis; UTS: urine toxicology screen; VAS: visual analog scale.

*conference abstract.

**Table 3:** Risk of bias assessment of randomized and non-randomized intervention studies.

**Revised Cochrane Risk-of-Bias Tool for Randomized Trials, Second Version (RoB 2)**									
**-**	**Confounding**	**Selection**	**Performance**	**Intervention Classification**	**Intervention ** **Deviation**	**Attrition**	**Detection**	**Reporting**	**Overall**
Batki *et al*., 2009*	-	High risk	Some concerns	-	-	Some concerns	Low risk	High risk	High risk
Bratu and Sopterean* 2014	-	High risk	High risk	-	-	Low risk	Low risk	High risk	High risk
Petrakis *et al*. 2004	-	Some concerns	Some concerns	-	-	Low risk	Low risk	Some concerns	Some concerns
Petrakis *et al*. 2005	-	Some concerns	Some concerns	-	-	Low risk	Low risk	Some concerns	Some concerns
Ralevski *et al*., 2011	-	Some concerns	High risk	-	-	Low risk	Low risk	Some concerns	Some concerns
Vasile *et al*., 2013	-	Some concerns	Some concerns	-	-	Low risk	Some concerns	High risk	High risk
**The Risk of Bias in Non-randomized Studies-of Interventions (ROBINS-I) Assessment Tool**									
Batki *et al*., 2007	High risk	Some concerns	-	Some concerns	Low risk	Some concerns	High risk	Some concerns	High risk
Batki *et al*. 2010*	High risk	High risk	-	Some concerns	Low risk	Some concerns	High risk	Some concerns	High risk
Maxwell & Shinderman, 2000	High risk	High risk	-	Some concerns	Low risk	High risk	High risk	Low risk	High risk
Mueser *et al*., 2003	High risk	High risk	-	Some concerns	Some concerns	High risk	High risk	Low risk	High risk
Vasile *et al*. 2014*	High risk	High risk	-	Some concerns	Low risk	Some concerns	High risk	Some concerns	High risk
Vasiliu *et al*. 2020	High risk	High risk	-	Some concerns	Low risk	High risk	Some concerns	Low risk	High risk

**Note:** -Not applicable for this type of study design.

*Note that for the sake of homogeneity, the ROBINS-I categories have been converted to RoB2 categories as follows: critical or serious risk= high risk; moderate risk = some concerns; low risk = low.

## LIST OF ABBREVIATIONS

AUD: Alcohol Use Disorder
CBT: Cognitive Behavioral Therapy
PD: Psychotic Disorders
PRISMA: Preferred Reporting Items for Systematic Reviews and Meta-Analyses
RCTs: Randomized Controlled Trials
SGOT: Serum Glutamic-Oxaloacetic Transaminase
SGPT: Serum Glutamic Pyruvic Transaminase
TL: Transaminase Levels

## STANDARDS OF REPORTING

PRISMA guidelines and methodology were followed.
