# Supplementary material for: Pharmacological Treatment of Alcohol use Disorder in Patients with Psychotic Disorders: A Systematic Review
Source: Curr Neuropharmacol. 2023 Mar 9;22(6):1129–43. doi: 10.2174/1570159X21666221229160300 (PMC10964102; doi:10.2174/1570159X21666221229160300)
Supplement: Supplementary file 1 [file CN-22-1129_SD1.pdf]

Supplementary Material

Pharmacological Treatment of Alcohol use Disorder in Patients with Psychotic Disorders: A Systematic Review

Niels Jørgen Rosenstand[1,6], Anette Søgaard Nielsen[1,2,3], Lotte Skøt[1], Simon Anhøj[1,2,3,4], Mikkel Højlund[5,6], Dorthe Grüner Nielsen[1,8] and Angelina Isabella Mellentin[1,2,3,7,*]

[1]Department of Clinical Research, Unit for Clinical Alcohol Research, University of Southern Denmark, Odense, Denmark; [2]Department of Psychiatry, Odense University Hospital, Region of Southern Denmark, Odense, Denmark; [3]Department of Clinical Research, Brain Research-Inter-Disciplinary Guided Excellence (BRIDGE), University of Southern Denmark, Odense, Denmark; [4]Department of Psychiatry, Region of Southern Denmark, Svendborg, Denmark; [5]Department of Public Health, Clinical Pharmacology, Pharmacy, and Environmental Medicine, University of Southern Denmark, Odense, Denmark; [6]Department of Psychiatry Aabenraa, Mental Health Services in the Region of Southern Denmark, Aabenraa, Denmark; [7]Research Unit for Telepsychiatry and E-Mental Health, Center for Telepsychiatry, Region of Southern Denmark, Odense, Denmark; [8]Drug Treatment Center Odense, Odense C, Denmark

1875-6190/24 $65.00+.00 **© 2024 Bentham Science Publishers**

Search Strategy

The main search was performed on three facets based on keywords related to “alcohol use disorder” (facet 1), “pharmacological treatment” (facet 2), and “psychotic disorder” (facet 3). Since possible subject headings varied for the different databases, we customized keywords according to the database. Within each facet, the subject headings were connected by OR, and the three facets were connected by AND (see table below). The subject headings used in the first Google Scholar search were: “alcohol use disorder”, “psychosis”, “disulfiram”, “naltrexone”, “nalmefene”, and “acamprosate”. In the second search, "schizophrenia” was added to the first search". In the third search, "schizoaffective" was added to the first search.

**Facet**	**Database**			
	**Ovid MEDLINE**	**Embase Classic+Embase**	**Cochrane Library**	**APA PsychInfo**
1	1. ”alcohol use disorder*”.mp.<br>2. aud.mp.<br>3. alcohol related disorder*.mp.<br>4. alcohol abuse.mp.<br>5. alcohol addiction.mp.<br>6. alcohol dependence.mp.<br>7. alcoholi*.mp.<br>8. exp alcoholism/	1. ”alcohol use disorder*”.mp.<br>2. aud.mp.<br>3. alcohol related disorder*.mp.<br>4. alcohol abuse.mp.<br>5. alcohol addiction.mp.<br>6. alcohol dependence.mp.<br>7. alcoholi*.mp.<br>8. exp alcoholism/<br>9. exp alcohol abuse/	1. ”alcohol use disorder*”.mp.<br>2. aud.mp.<br>3. alcohol related disorder*.mp.<br>4. alcohol abuse.mp.<br>5. alcohol addiction.mp.<br>6. alcohol dependence.mp.<br>7. alcoholi*.mp.<br>8. exp alcoholism/	1. ”alcohol use disorder*”.mp.<br>2. aud.mp.<br>3. alcohol related disorder*.mp.<br>4. alcohol abuse.mp.<br>5. alcohol addiction.mp.<br>6. alcohol dependence.mp.<br>7. alcoholi*.mp.<br>8. exp alcoholism/<br>9. exp alcohol abuse/
2	9. disulfiram.mp.<br>10. naltrexone.mp.<br>11. acamprosate.mp.<br>12. nalmefene.mp.<br>13. disulfiram/<br>14. naltrexone/<br>15. acamprosate/<br>16. Alcohol Deterrents/	10. disulfiram.mp.<br>11. naltrexone.mp.<br>12. acamprosate.mp.<br>13. nalmefene.mp.<br>14. disulfiram/<br>15. naltrexone/<br>16. acamprosate/<br>17. nalmefene/	9. disulfiram.mp.<br>10. naltrexone.mp.<br>11. acamprosate.mp.<br>12. nalmefene.mp.<br>13. exp disulfiram/<br>14. exp naltrexone/<br>15. exp acamprosate/	10. disulfiram.mp.<br>11. naltrexone.mp.<br>12. acamprosate.mp.<br>13. nalmefene.mp.<br>14. disulfiram/<br>15. naltrexone/<br>16. acamprosate/<br>17. exp Alcohol Treatment/
3	17. psychosis.mp.<br>18. psychoses.mp.<br>19. psychotic.mp.<br>20. schizophrenia.mp.<br>21. schizoid*.mp.<br>22. schizophrenic disorder*.mp.<br>23. schizophreniform.mp.<br>24. schizoaffective.mp.<br>25. schizoaffective psychosis.mp.<br>26. exp schizophrenia/<br>27. Psychotic Disorders/	18. psychosis.mp.<br>19. psychoses.mp.<br>20. psychotic.mp.<br>21. schizophrenia.mp.<br>22. schizoid*.mp.<br>23. schizophrenic disorder*.mp.<br>24. schizophreniform.mp.<br>25. schizoaffective.mp.<br>26. schizoaffective psychosis.mp.<br>27. exp psychosis/<br>28. exp schizophrenia/<br>29. schizoidism/	16. psychosis.mp.<br>17. psychotic.mp.<br>18. schizophrenia.mp.<br>19. schizoid*.mp.<br>20. schizophrenic disorder*.mp.<br>21. schizophreniform.mp.<br>22. schizoaffective.mp.<br>23. schizoaffective psychosis.mp.<br>24. exp Psychotic Disorders/	18. psychosis.mp.<br>19. psychoses.mp.<br>20. psychotic.mp.<br>21. schizophrenia.mp.<br>22. schizoid*.mp.<br>23. schizophrenic disorder*.mp.<br>24. schizophreniform.mp.<br>25. schizoaffective.mp.<br>26. schizoaffective psychosis.mp.<br>27. exp psychosis/<br>28. exp schizophrenia/<br>29. Schizoaffective Disorder
